# Variations in Treatment Prescription and Survival Outcomes Between Older and Younger Patients With Mucosal Head and Neck Squamous Cell Carcinoma Treated With Radiotherapy

**DOI:** 10.1002/hed.70310

**Published:** 2026-05-05

**Authors:** Farhannah Aly, Joseph Descallar, Purnima Sundaresan, Alexis Andrew Miller, Lois Holloway, Shalini Vinod

**Affiliations:** ^1^ Southwest Sydney Clinical Campus University of New South Wales Sydney Australia; ^2^ Liverpool and Macarthur Cancer Therapy Centres, SWSLHD Sydney Australia; ^3^ Ingham Institute for Applied Medical Research Sydney Australia; ^4^ Sydney West Radiation Oncology Network WSLHD Sydney Australia; ^5^ Sydney Medical School University of Sydney Sydney Australia; ^6^ Illawarra Cancer Care Centre ISLHD Wollongong Australia; ^7^ Institute of Medical Physics, School of Physics University of Sydney Sydney Australia

**Keywords:** geriatric oncology, head and neck squamous cell carcinoma, radiotherapy, retrospective studies, survival

## Abstract

**Purpose:**

To investigate treatment variations and outcomes between older (≥ 70 years) and younger (< 70 years) patients with mucosal head and neck squamous cell carcinoma (mHNSCC) treated with radiotherapy.

**Methods:**

A multicenter retrospective review of patients diagnosed from 2010 to 2018 was conducted. Patient, tumor, and treatment data were collected, including overall survival (OS) and progression‐free survival (PFS). Multivariable analyses examined factors influencing standard‐of‐care (SOC) treatment, OS, and PFS.

**Results:**

Of 1553 patients, 432 patients were ≥ 70 years. Older patients were significantly less likely to receive curative treatment, SOC, or concurrent systemic therapy. Age, marital status, ECOG status, stage, and subsite influenced SOC treatment. Three‐year OS and PFS were lower in older patients. ECOG status, stage, subsite, and SOC treatment were significantly associated with OS and PFS. Older age and smoking status were significantly associated with lower OS but not PFS.

**Conclusions:**

Older patients should be offered SOC treatment where appropriate for maximal disease control and survival.

## 1 Introduction

Treatment guidelines based on clinical trial results provide evidence‐based recommendations to guide clinical practice. Despite over 50% of patients newly diagnosed with cancer being ≥ 65 years of age, older patients are frequently underrepresented in clinical trials [1, 2, 3]. Those older patients who meet the strict eligibility criteria and participate in clinical trials tend to be the “fit old,” meaning that extrapolation of results to frailer patients can be difficult. Older patients with mucosal head and neck squamous cell carcinoma (mHNSCC) are a particular group that presents complex challenges in management given the multimodality treatment approaches and the involvement of organs whose form and function are vitally important to quality of life.

Older patients with similar chronological age can differ greatly in their biological age. Older patients may have comorbidities and poorer performance status, affecting their noncancer‐related life expectancy. The benefit derived from the standard‐of‐care (SOC) treatment may be limited, and consideration of treatment toxicity and quality of life may be of greater importance than for younger patients [4]. Reduced age‐related physiological function, comorbidities (e.g., impaired hearing and renal impairment), and reduced functional reserve also contribute to increased adverse treatment effects, requiring a personalized treatment approach [5].

Standard treatment approaches depend on the site and stage of the primary cancer, with multiple treatment guidelines available [6, 7, 8]. Common variations from SOC treatment utilized for older or frailer patients with mHNSCC include substitution of carboplatin for cisplatin, use of cetuximab instead of chemotherapy, altered RT fractionation regimens (hypofractionation), primary RT instead of surgery, and palliative‐intent treatment rather than curative treatment.

There is limited literature specifically reporting on treatment approaches and outcomes of older patients with mHNSCC. Several single‐institution retrospective studies observed that older patients with HNSCC were more likely to receive deviations from SOC treatment approaches than younger patients [9, 10, 11, 12]. There is mixed evidence in terms of survival outcomes following RT for older patients when compared to younger patients. Where poorer survival was observed in older patients, a greater proportion of older patients received nonstandard treatment approaches compared to younger patients [11, 13, 14]. Other studies demonstrating similar survival outcomes between older and younger age cohorts [12, 15] suggest aiming for SOC treatment where possible in older patients is warranted.

Many older studies are in historical treatment cohorts. With evolution in the management of mHNSCC in recent years, analysis of contemporary data is important to understand current variations in the management of elderly patients. The aim of this study was to investigate the characteristics, patterns of care, and survival outcomes of older (≥ 70 years) versus younger (18–69 years) patients with mHNSCC treated with radiotherapy in a contemporary Australian cohort.

## 2 Materials and Methods

### 2.1 Participants

This was a multicenter retrospective study of patients treated with radiotherapy, as part of their mHNSCC treatment, at six hospitals. Routine practice at these institutions is for all patients with mHNSCC to be discussed at a multidisciplinary team (MDT) meeting. The participating institutions treat patients from diverse geographical locations (metropolitan and regional areas) and cultural backgrounds (although detailed demographic data was unavailable for analysis). The study population, identified from oncology information systems, consisted of adult patients with a new diagnosis of histologically proven mHNSCC and identifiable site of origin, diagnosed from 2010 to 2018, allowing sufficient follow‐up for reporting outcomes. Patients with squamous cell carcinoma (SCC) arising from skin or salivary glands were excluded. Patients were treated with RT, with either curative or palliative intent, in a primary or adjuvant setting. The older cohort was defined as aged 70 years or older, consistent with European Organization for Research and Treatment of Cancer (EORTC) and International Society of Geriatric Oncology (SIOG) definitions [16, 17]. This threshold reflects the increasing incidence of age‐related physiological decline and treatment‐related vulnerability from approximately 70 years onward and its common use as a reference point in oncology clinical trials [18]. Recent SIOG guidance also acknowledges the lack of a consensus definition, with some studies applying a lower threshold of 65 years [19]. Ethics board approval was obtained for this study.

### 2.2 Data Extraction

Data were obtained from oncology information systems (MOSAIQ or ARIA) and hospital electronic medical records. Data unavailable in structured format were manually extracted from patient records where possible. Patient data comprised age at diagnosis, sex, marital status, smoking status, smoking pack year history, and Eastern Cooperative Oncology Group (ECOG) performance status. Tumor subsite, stage, grade, and p16 status were extracted. Treatment data included treatment intent (curative or palliative), systemic therapy agent, RT dose (including biologically effective dose [BED] for palliative treatments), RT completion, overall treatment time, and whether patients received SOC treatment. For palliative treatments, the BED was calculated as (total delivered dose) × (1 + [fraction dose/(α/β)]), with α/β = 10. Tumor stage was recorded in accordance with the American Joint Committee on Cancer (AJCC) version 7 classification. For two participating centers, where ECOG performance status was not recorded, it was inferred where possible by a radiation oncologist using the clinical history recorded at the time of diagnosis. SOC for curative intent was defined according to Australian management guidelines for head and neck cancer (Table S1) [7]. SOC treatment assignment for this study was performed by reviewing individual patient records.

### 2.3 Statistical Analysis

Descriptive statistics of patient, tumor, and treatment variables were reported. Differences between age groups were assessed using Fisher's exact test (categorical variables) and a negative binomial model (smoking pack years). A multivariable logistic regression model was constructed to determine variables influencing SOC assignment. Variables were selected for inclusion based on clinical judgment, and all were included in both univariable and multivariable modeling. Hosmer–Lemeshow goodness of fit testing was performed to test model assumptions. Overall survival (OS) and progression‐free survival (PFS) were measured from diagnosis to date of outcome or right‐censored at last follow‐up. Progression was defined as tumor recurrence after complete response or progression if there was no complete response to RT. Kaplan–Meier survival analysis of survival outcomes was undertaken. Differences in OS and PFS between age cohorts were assessed using the log‐rank test. Multivariable Cox proportional hazards models were used to assess the relationship of variables to survival outcomes. Schoenfeld residual tests were examined to test the proportionality assumption. *p* < 0.05 were considered statistically significant, with all *p* values being two‐sided. Missing data for marital status, ECOG, smoking status, and SOC assignment were imputed using the multiple imputation by chained equations (MICE) method in the “mice” R package [20]. Sensitivity testing for management of missing data was performed to ensure results were accurate. A combined analysis was performed of all institutions, and results were adjusted for institution differences during modeling. Statistical analysis was conducted using R Statistical Software (v 4.3.2).

## 3 Results

### 3.1 Pretreatment Characteristics

A total of 1553 patients met eligibility criteria; 1121 (72.2%) < 70 years, 432 (27.8%) aged 70+ years (Table 1). The median age and interquartile range (IQR) were 59.2 (52.5–64.5) years for younger and 76.2 (72.8–80.6) years for older patients. The older cohort had a significantly greater proportion of females (*p* < 0.001), unpartnered status (*p* = 0.005), and former smokers (*p* < 0.001) than the younger cohort. Older patients were less likely to have good ECOG performance status (*p* < 0.001) and oropharyngeal primaries (*p* < 0.001) compared to younger patients. The median (IQR) smoking pack‐year history was 35.0 (20.0–45.0) and 40.0 (24.2–50.0) years for younger and older patients, respectively.

**TABLE 1:** Pretreatment patient and tumor characteristics for the full cohort and by age group.

Characteristic, *n* (%)	Full cohort (*n* = 1553)	< 70 years (*n* = 1121)	≥ 70 years (*n* = 432)	*p*
Participating center				0.939
Centre A	557 (35.9)	399 (35.6)	158 (36.6)	
Centre B	378 (24.3)	274 (24.4)	104 (24.1)	
Centre C	618 (39.8)	448 (40.0)	170 (39.4)	
Sex				**< 0.001**
Male	1231 (79.3)	918 (81.9)	313 (72.5)	
Female	322 (20.7)	203 (18.1)	119 (27.5)	
Marital status				**0.005**
Partnered	867 (55.8)	651 (58.1)	216 (50.0)	
Unpartnered	609 (39.2)	423 (37.7)	186 (43.1)	
Missing	77 (5.0)	47 (4.2)	30 (6.9)	
Smoking status				**< 0.001**
Never	320 (20.6)	234 (20.9)	86 (19.9)	
Former	580 (37.3)	381 (34.0)	199 (46.1)	
Current	503 (32.4)	407 (36.3)	96 (22.2)	
Missing	150 (9.7)	99 (8.8)	51 (11.8)	
Median smoking pack years (IQR)	38.0 (20.0–50.0)	35.0 (20.0–45.0)	40.0 (24.2–50.0)	**< 0.001**
Smoking pack‐years category^a^				
< 10	393 (25.3)	294 (26.2)	99 (22.9)	0.091
≥ 10	799 (51.4)	582 (51.9)	217 (50.2)	
Missing	361 (23.2)	245 (21.9)	116 (26.9)	
ECOG				**< 0.001**
0	664 (42.8)	555 (49.5)	109 (25.2)	
1	303 (19.5)	191 (17.0)	112 (25.9)	
2+	127 (8.2)	54 (4.8)	73 (16.9)	
Missing	459 (29.6)	321 (28.6)	138 (31.9)	
Tumor sites				**< 0.001**
Oropharynx	621 (40.0)	504 (45.0)	117 (27.1)	
Oral cavity	321 (20.7)	212 (18.9)	109 (25.2)	
Larynx	294 (18.9)	166 (14.8)	128 (29.6)	
Nasopharynx	148 (9.5)	133 (11.9)	15 (3.5)	
Hypopharynx	117 (7.5)	71 (6.3)	46 (10.6)	
Paranasal sinus	30 (1.9)	22 (2.0)	8 (1.9)	
Other	22 (1.4)	13 (1.2)	9 (2.1)	
Tumor stage				**< 0.001**
I	90 (5.8)	48 (4.3)	42 (9.7)	
II	169 (10.9)	106 (9.5)	63 (14.6)	
III	292 (18.8)	216 (19.3)	76 (17.6)	
IVA/B	811 (52.2)	616 (55.0)	195 (45.1)	
IVC	74 (4.8)	53 (4.7)	21 (4.9)	
Unknown^b^	117 (7.5)	82 (7.3)	35 (8.1)	
Grade (excluding NPX)				0.765
1	100 (7.1)	68 (6.9)	32 (7.7)	
2	420 (29.9)	295 (29.9)	125 (30.0)	
3	352 (25.1)	252 (25.5)	100 (24.0)	
4 (undifferentiated)	6 (0.4)	3 (0.3)	3 (0.7)	
Missing	527 (37.5)	370 (37.4)	157 (37.6)	
p16 status (OPX)				0.107
Positive	291 (40.3)	246 (48.8)	45 (38.5)	
Negative	80 (12.9)	64 (12.7)	16 (13.7)	
Missing	250 (40.3)	194 (38.5)	56 (47.9)	

*Note:* The bold values represent statistically significant *p* values.

Abbreviations: IQR, interquartile range; NPX, nasopharynx cancer; OPX, oropharynx cancer.

^a^ Never smokers are categorized as < 10 pack years.

^b^ TNM stage is incompletely recorded.

### 3.2 Treatment Approach

**FIGURE 1:**
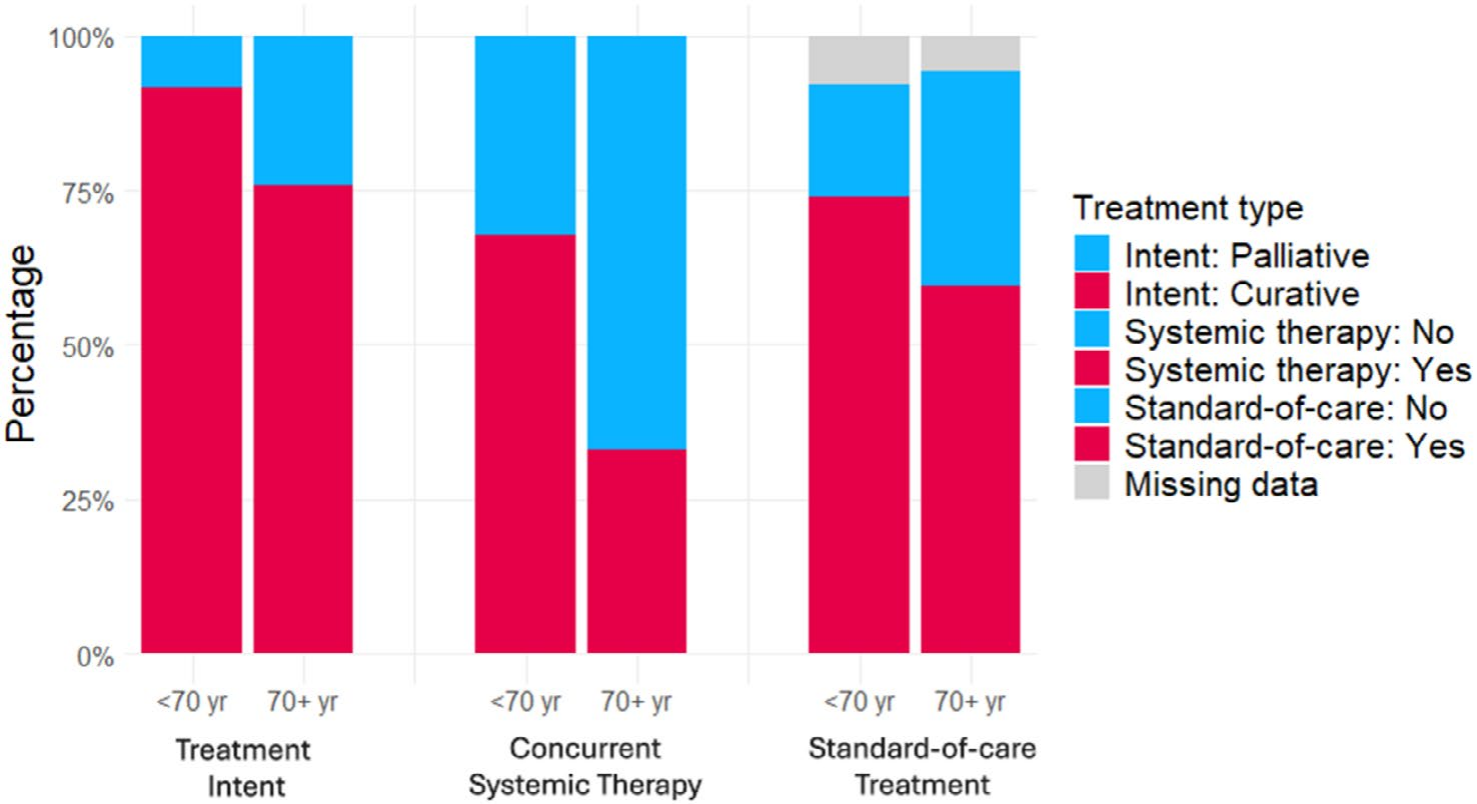
Treatment type by age category. [Color figure can be viewed at wileyonlinelibrary.com]

Fewer older patients received curative treatment approaches, 75.7% versus 91.6% (*p* < 0.001) (Table 2). For those receiving curative RT, fewer older patients were prescribed systemic therapy (*p* < 0.001) (Figure 1). For patients receiving concurrent systemic therapy, older patients were less likely to receive cisplatin (*p* < 0.001 primary RT, *p* = 0.014 adjuvant RT). There were no significant differences between older and younger patients treated with curative intent in prescribed RT dose (early glottis cancers were excluded from this analysis, as RT dose < 66 Gy may be acceptable as SOC treatment). For early glottis cancers, 61.4% of younger patients were prescribed altered fractionation regimens (e.g., hypofractionation or twice‐daily fractionation) compared to 44.9% of older patients, though the difference was statistically nonsignificant (*p* = 0.088). Prescription of SOC treatment was significantly lower in older patients than younger patients, 59.6% versus 74.0% (*p* < 0.001). Nonstandard treatments included omission of concurrent systemic therapy, use of cetuximab or carboplatin as concurrent systemic therapy, nonstandard RT dose, and primary RT where surgery is considered SOC (Table 2). Older patients were more likely to have concurrent systemic therapy omitted, 38.9% versus 8.6%, and less likely to be prescribed carboplatin or cetuximab, 41.6% versus 61.5%, than younger patients (*p* < 0.001). Differences in RT completion rates between the age cohorts (older 86.2%, younger 89.7%) were not statistically significant (*p* = 0.06). For patients receiving primary RT, the median overall treatment time for both cohorts was 45 days, and for adjuvant RT, 39 days. For patients receiving palliative‐intent RT, there were no significant differences in proportions prescribed high (BED ≥ 50 Gy) or low (BED < 50 Gy) dose of RT between the age cohorts.

**TABLE 2:** Treatment approach for the full cohort and by age groups.

Characteristic, *n* (%)	Full cohort (*n* = 1553)	< 70 years (*n* = 1121)	≥ 70 years (*n* = 432)	*p*
Treatment intent				**< 0.001**
Curative	1354 (87.2)	1027 (91.6)	327 (75.7)	
Palliative	199 (12.8)	94 (8.4)	105 (24.3)	
Concurrent systemic therapy^a^				**< 0.001**
Yes	804 (59.4)	696 (67.8)	108 (33.0)	
No	550 (40.6)	331 (32.2)	219 (67.0)	
Treatment type (primary RT)^a^				**< 0.001**
Primary RT + systemic therapy	719 (73.4)	620 (82.9)	99 (42.9)	
Primary RT alone	260 (26.6)	128 (17.1)	132 (57.1)	
Treatment type (adjuvant RT)^a^				**< 0.001**
Adjuvant RT + systemic therapy	85 (22.7)	76 (27.2)	9 (9.4)	
Adjuvant RT alone	290 (77.3)	203 (72.8)	87 (90.6)	
Concurrent systemic therapy type^a^ (primary RT)				**< 0.001**
Cisplatin	493 (68.3)	449 (72.1)	44 (44.4)	
Cetuximab	114 (15.8)	83 (13.3)	31 (31.3)	
Carboplatin	61 (8.4)	45 (7.2)	16 (16.2)	
Missing info on agent	54 (7.5)	46 (7.4)	8 (8.1)	
Concurrent systemic therapy type^a^ (adjuvant RT)				**0.014**
Cisplatin	63 (73.3)	60 (77.9)	3 (33.3)	
Cetuximab	10 (11.6)	6 (7.8)	4 (44.4)	
Carboplatin	4 (4.7)	3 (3.9)	1 (11.1)	
Missing info on agent	9 (10.5)	8 (10.4)	1 (11.1)	
Prescribed RT dose^a^, ^b^ (primary RT)				0.064
≥ 66 Gy	806 (92.2)	640 (92.5)	166 (91.2)	
< 66 Gy	26 (3.0)	16 (2.3)	10 (5.5)	
Missing	42 (4.8)	36 (5.2)	6 (3.3)	
RT fractionation for stage I‐II glottis cancer				
Standard fractionation	47 (44.3)	21 (36.8)	26 (53.1)	0.158
Altered fractionation	57 (53.8)	35 (61.4)	22 (44.9)	
Missing	2 (1.9)	1 (1.8)	1 (2.0)	
Prescribed RT dose^a^ (adjuvant RT)				0.088
≥ 60 Gy	335 (88.9)	249 (88.6)	86 (89.6)	
< 66 Gy	13 (3.4)	7 (2.5)	6 (6.2)	
Missing	29 (7.7)	25 (8.9)	4 (4.2)	
Prescribed standard of care treatment^a^				**< 0.001**
Yes	955 (70.5)	760 (74.0)	195 (59.6)	
No	300 (22.2)	187 (18.2)	113 (34.6)	
Missing	99 (7.3)	80 (7.8)	19 (5.8)	
Nonstandard prescription				**< 0.001**
No systemic therapy	60 (20.0)	16 (8.6)	44 (38.9)	
Cetuximab or Carboplatin	162 (54.0)	115 (61.5)	47 (41.6)	
Nonstandard RT dose	25 (8.3)	15 (8.0)	10 (8.8)	
Primary RT use where surgery SOC	36 (12.0)	26 (13.9)	10 (8.8)	
Other	17 (5.7)	15 (8.0)	2 (1.8)	
RT completion^a^, ^c^				0.060
Yes	1203 (88.8)	921 (89.7)	282 (86.2)	
No	56 (4.1)	35 (3.4)	21 (6.4)	
Missing info	95 (7.0)	71 (6.9)	24 (7.3)	
Delivered RT dose (palliative RT)				0.737
High dose (BED ≥ 50 Gy)	79 (41.8)	36 (40.4)	43 (43.0)	
Low dose (BED < 50 Gy)	100 (52.9)	47 (52.8)	53 (55.0)	
Missing	10 (5.3)	6 (6.7)	4 (4.0)	

*Note:* The bold values represent statistically significant *p* values.

Abbreviation: SOC, standard of care.

^a^ Curative management patients only.

^b^ Excluding early glottis tumors, where it may be acceptable to prescribe less than 66 Gy as standard of care treatment.

^c^ Delivered RT dose ≥ prescribed RT dose.

Table 3 presents the multivariable analysis of clinical variables associated with SOC treatment prescription (univariable analysis in Table S2). Older age (OR = 0.366, 95% CI: 0.262–0.511; *p* < 0.001), unpartnered marital status (OR = 0.722, 95% CI: 0.529–0.985; *p* = 0.04), ECOG status 1 (OR = 0.625, 95% CI 0.444–0.879; *p* = 0.007) or 2+ (OR = 0.390, 95% CI: 0.231–0.658; *p* < 0.001), and tumor AJCC Stage III (OR = 0.206, 95% CI: 0.086–0.494; *p* < 0.001), IVA/B (OR = 0.182, 95% CI: 0.077–0.428; *p* < 0.001), and IVC (OR = 0.014, 95% CI: 0.002–0.082; *p* < 0.001) were significantly associated with being prescribed nonstandard treatment (Table 3). The oral cavity subsite was significantly associated with being prescribed SOC treatment (OR = 2.642, 95% CI: 1.415–4.933; *p* = 0.002). The Hosmer–Lemeshow goodness of fit test indicated adequate model fit.

**TABLE 3:** Multivariable analysis of variables for standard‐of‐care treatment prescription, overall survival, and progression‐free survival.

		Standard of care treatment			Overall survival			Progression‐free survival		
Variable	Strata	OR	95% CI	*p*	HR	95% CI	*p*	HR	95% CI	*p*
Age	< 70 years	Ref			Ref			Ref		
	≥ 70 years	0.366	0.262–0.511	**< 0.001**	1.617	1.304–2.005	**< 0.001**	1.163	0.908–1.488	0.232
Sex	Female	Ref			Ref			Ref		
	Male	0.914	0.624–1.341	0.647	1.027	0.812–1.299	0.826	0.967	0.744–1.258	0.803
Marital status	Partnered	Ref			Ref			Ref		
	Unpartnered	0.722	0.529–0.985	**0.040**	1.055	0.877–1.269	0.573	0.896	0.720–1.116	0.328
ECOG status	0	Ref			Ref			Ref		
	1	0.625	0.444–0.879	**0.007**	1.529	1.243–1.880	**< 0.001**	1.388	1.091–1.766	**0.008**
	2+	0.390	0.231–0.658	**< 0.001**	2.948	2.132–4.078	**< 0.001**	1.870	1.225–2.853	**0.005**
Smoking status	Current	Ref			Ref			Ref		
	Former	0.962	0.682–1.356	0.824	0.659	0.525–0.827	**0.001**	0.857	0.671–1.095	0.218
	Never	0.991	0.638–1.539	0.969	0.589	0.439–0.789	**0.001**	0.764	0.554–1.052	0.101
Tumor site	Hypopharynx	Ref			Ref			Ref		
	Oropharynx	1.140	0.663–1.959	0.635	0.701	0.505–0.972	**0.034**	0.518	0.358–0.750	**0.001**
	Oral cavity	2.642	1.415–4.933	**0.002**	1.271	0.890–1.816	0.188	1.091	0.741–1.607	0.658
	Larynx	1.519	0.823–2.802	0.182	0.921	0.644–1.318	0.653	0.669	0.440–1.015	0.060
	Nasopharynx	1.019	0.493–2.107	0.960	0.634	0.396–1.014	0.058	0.710	0.433–1.167	0.178
	Other	1.271	0.501–3.223	0.613	0.976	0.535–1.781	0.938	0.900	0.474–1.708	0.747
Tumor stage	I	Ref			Ref			Ref		
	II	0.507	0.198–1.302	0.158	1.517	0.888–2.592	0.131	1.317	0.739–2.347	0.352
	III	0.206	0.086–0.494	**< 0.001**	1.805	1.108–2.939	**0.019**	1.404	0.810–2.433	0.227
	IVA/B	0.182	0.077–0.428	**< 0.001**	2.483	1.544–3.992	**< 0.001**	2.212	1.301–3.761	**0.004**
	IVC	0.014	0.002–0.082	**< 0.001**	1.711	0.577–5.074	0.333	2.056	0.716–5.905	0.182
SOC	Yes	NA			Ref			Ref		
	No	NA			0.764	0.615–0.948	**0.015**	0.608	0.480–0.771	**< 0.001**

*Note:* The bold values represent statistically significant *p* values.

Abbreviations: CI, confidence interval; OR, odds ratio; Ref, reference variable; SOC, standard of care treatment.

### 3.3 Patient Outcomes

The median follow‐up duration was 54.1 months for curative patients and 7.1 months for palliative patients. There were a total of 695 deaths, 449 in the younger cohort and 246 in the older cohort. In the curatively treated population, of a total of 538 deaths, 370 and 168 were in the younger and older cohorts, respectively. Table 4 presents causes of death and initial recurrence site. Older patients had a significantly higher proportion of noncancer‐related deaths compared to younger patients (26.8% vs. 17.0%; *p* = 0.032). A total of 409 recurrences were observed in the curative cohort. There were no significant differences between the older and younger patients in initial recurrence site (*p* = 0.191), deaths within 30 days of RT completion (*p* = 0.346), or cause of death in the palliative cohort (*p* = 0.086; Table S3).

**TABLE 4:** Causes of death, death within 30 days of RT completion, and initial recurrence site for the full cohort and by age group for the curatively treated population.

	Curative cohort (*n* = 1354)	< 70 years (*n* = 1027)	≥ 70 years (*n* = 432)	*p* ^a^
Deaths, *n*	538	370	168	
Cause of death, *n* (%)				**0.032**
Cancer‐related death	333 (61.9)	236 (63.8)	97 (57.7)	
Non‐cancer death	108 (20.1)	63 (17.0)	45 (26.8)	
Unknown cause of death	97 (18.0)	71 (19.2)	26 (15.5)	
Death within 30 days of RT completion, *n* (%)				0.346
Yes	22 (4.5)	13 (3.8)	9 (5.9)	
No	469 (95.5)	326 (96.2)	143 (94.1)	
Recurrences, *n*	409	301	108	
Initial recurrence site, *n* (%)				0.191
Locoregional only	227 (55.5)	159 (52.8)	68 (63.0)	
Distant only	104 (25.4)	82 (27.2)	22 (20.4)	
Locoregional and distant	78 (19.1)	60 (19.9)	18 (16.7)	

*Note:* The bold values represent statistically significant *p* values.

^a^ Fishers exact test.

**FIGURE 2:**
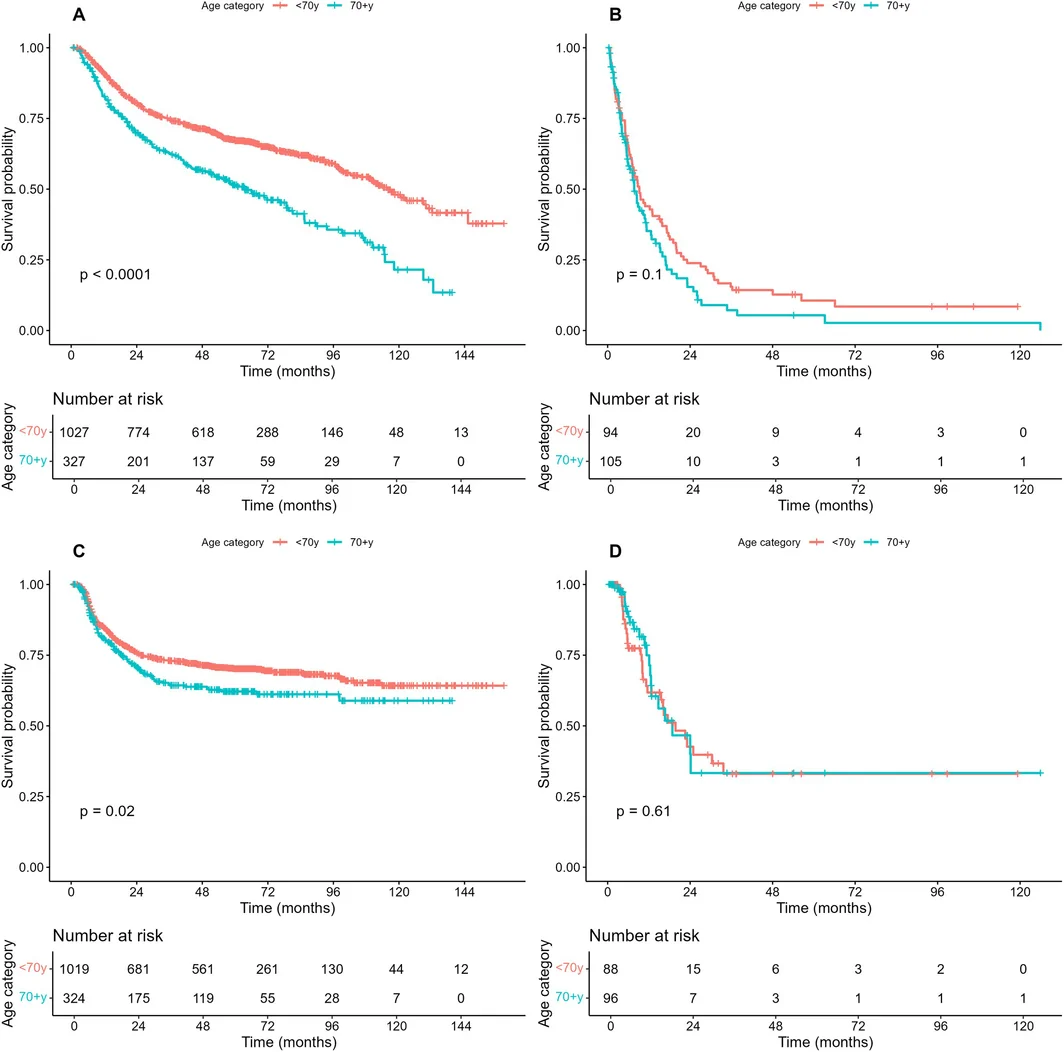
Overall survival for patients treated with (A) curative treatment and (B) palliative treatment; and progression‐free survival for patients treated with (C) curative treatment and (D) palliative treatment. [Color figure can be viewed at wileyonlinelibrary.com]

Median OS for younger patients treated with curative intent was significantly higher than for older patients (*p* < 0.001): 115.6 months (95% CI: 105.8–132.1) versus 63.5 months (95% CI: 51.3–79.7), respectively (Figure 2A). Three‐ and five‐year OS for patients receiving curative treatment was 74.6% (95% CI: 71.9–77.4) and 67.3% (95% CI: 64.4–70.4) for the younger cohort, 62.6% (95% CI: 57.4–68.3) and 51.5% (95% CI: 45.9–57.7) for the older cohort. Multivariable Cox proportional hazard modeling demonstrated that older age (HR = 1.617, 95% CI: 1.304–2.005; *p* < 0.001), ECOG 1 (HR = 1.529, 95% CI: 1.243–1.880; *p* < 0.001) and 2+ (HR = 2.948, 95% CI: 2.132–4.078; *p* < 0.001) performance status, and tumor AJCC Stage III (HR = 1.805, 95% CI: 1.108–2.939; *p* = 0.019) and IVA/B (HR = 2.483, 95% CI: 1.544–3.992; *p* < 0.001) were significantly associated with worse OS; former smoking status (HR = 0.659, 95% CI: 0.525–0.827; *p* = 0.001), never smoking status (HR = 0.589, 95% CI: 0.439–0.789; *p* = 0.001), oropharynx site (HR = 0.701, 95% CI: 0.505–0.972; *p* = 0.034), and SOC treatment prescription (HR = 0.764, 95% CI: 0.615–0.948; *p* = 0.015) were significantly associated with improved OS (Table 3; Table S2).

The older cohort had significantly worse PFS than the younger cohort, *p* = 0.02 (Figure 2C). The median PFS was not reached. Three‐ and five‐year PFS were 73.0% (95% CI: 70.2–75.8) and 70.2% (95% CI: 67.4–73.3) for the younger cohort and for the older cohort, 64.8% (95% CI: 59.3–70.8) and 62.2% (95% CI: 56.5–68.4). In multivariable analysis (Table 3), ECOG 1 (HR = 1.388, 95% CI: 1.091–1.766, *p* = 0.008) and 2+ performance status (HR = 1.870, 95% CI: 1.225–2.853; *p* = 0.005) and tumor AJCC stage IVA/B (HR = 2.212, 95% CI: 1.301–3.761; *p* = 0.004) were significantly associated with worse PFS; oropharynx subsite (HR = 0.518, 95% CI: 0.358–0.750; *p* = 0.001) and SOC treatment prescription (HR = 0.608, 95% CI: 0.480–0.771; *p* < 0.001) were significantly associated with improved PFS. While age was a significant variable associated with PFS on univariable analysis (Table S2), it was not significant on multivariable analysis. Visual inspection of Schoenfeld residual plots for OS and PFS demonstrated that the proportional hazards assumptions were satisfied.

Interaction between age group and treatment strategy was assessed by incorporating an interaction term between age group and treatment strategy within the multivariable models for OS and PFS. This analysis assessed effect modification, specifically whether the association between SOC treatment and outcomes varied by age. The interaction terms were not statistically significant for either OS (*p* = 0.750) or PFS (*p* = 0.122), indicating no evidence that the effect of SOC treatment differs between younger and older patients within this cohort (Table S4).

Since HPV‐related oropharyngeal cancers demonstrate a distinct epidemiological profile, a subset analysis for patients with oropharyngeal cancer and for whom p16 status was available (*n* = 354) was performed. After accounting for p16 status, age remained associated with improved OS, but not PFS. For the OS analysis, the HR for age was 2.439 (95% CI: 1.459–4.078), consistent with the analysis in the full cohort (Table S5). For PFS, we did not find an association between age group and p16 status (*p* = 0.069), also consistent with the analysis in the full cohort.

For patients treated with palliative intent, there were no significant differences in median OS and PFS for the age cohorts (Figure 2B,D). Median OS was 9.2 months (95% CI: 7.1–15.7) and 7.7 months (95% CI: 6.0–11.3) for younger and older patients, respectively, with median PFS being 19.7 months (95% CI: 11.6–NA) and 18.8 months (95% CI: 12.7–NA).

## 4 Discussion

This multicenter study investigated variations in pretreatment characteristics, patterns of care, and outcomes in a large (*n* = 1553) contemporary multicenter cohort of patients with mHNSCC. Older patients with mHNSCC were less likely to be prescribed SOC treatment, curative intent treatment, and systemic therapies. Where systemic therapy was prescribed for older patients, they were less likely to receive cisplatin. Our findings are consistent with other studies reporting that older patients are less likely to receive concurrent chemoradiotherapy than younger patients with mHNSCC. Juarez et al. [10] reported 23% of older patients (≥ 70 years) received chemoradiotherapy, Sommers et al. [14] reported 8.4% and Huang et al. [11] reported 6% for patients ≥ 75 years. In contrast, 33% of older patients in our population cohort received concurrent chemoradiotherapy, greater than reported in these historical cohorts. A multicenter cohort study [21] reported that 77.6% received concomitant chemoradiotherapy, a much larger proportion than our cohort. However, their age inclusion criteria were ≥ 65 years; younger patients in their cohort may not have had aging‐related physiological decline and comorbidities that would preclude the use of systemic therapy.

We found that prescription of SOC treatment was associated with younger patient age, as well as lower tumor stage, partnered marital status, and good ECOG performance status. Lack of social support and poor performance status may affect the ability to cope with prescribed treatment [17]. Age‐related physiological alterations and the presence of comorbidities can decrease tolerance to chemotherapy [17, 22]. Older patients may also potentially choose fewer toxic treatments and prioritize quality of life over disease‐specific survival [23]. These factors would be considered when prescribing treatment and would explain the lower rates of SOC treatment prescription observed in the older cohort. The oral cavity site was associated with SOC treatment prescription in our curatively treated population. This reflects clinical practice, where if a patient were not fit for surgery, then they would usually be treated with palliative intent, as curative intent nonsurgical treatment (i.e., chemoradiotherapy) is associated with significant treatment‐related toxicity + morbidity.

In this study, older patients experienced worse OS and PFS. Older age, nonstandard treatment prescription, smoking, poor ECOG performance status, and advanced tumor stage were significantly associated with worse OS. Nonstandard treatment prescription, poor ECOG performance status, and advanced tumor stage were associated with worse PFS on multivariable analysis. Older age was associated with worse OS but not PFS, suggesting that noncancer‐related mortality contributed to the inferior OS in the older cohort. This is supported by our finding of a significantly higher proportion of noncancer‐related deaths in the older population compared to the younger population. Older patients, smokers (former or current), and those with worse ECOG status may have other comorbidities, which contribute to increased noncancer‐related mortality. Smoking history may also be a surrogate marker for non‐HPV‐related cancers, which are usually associated with poorer outcomes. SOC treatment prescription was significantly associated with greater PFS and OS after accounting for the other clinical variables. Oropharyngeal cancers were significantly associated with better OS and PFS, which may be explained by the better prognosis of p16‐positive tumors of that site.

Our findings on OS and PFS are consistent with other studies [12, 15], including the association of a worse ECOG performance score with inferior OS. Where studies observed poorer survival in older patients [11, 13, 14], older patients received more nonstandard treatment approaches compared to younger patients, which may have affected outcomes. Rühle et al. reported that chemoradiotherapy was associated with longer OS and PFS than radiotherapy alone, with survival benefit seen in patients up to 80 years of age. These results support our findings that SOC treatment (which included chemoradiotherapy) was associated with improved outcomes. Aiming for SOC treatment approaches is warranted in older patients where possible, especially in robust older patients, though treatment decisions should consider biological age (rather than chronological age), comorbidities, functional status, social supports, and patient preferences through a shared decision‐making process.

Frameworks for developing a personalized treatment plan in older patients with cancer have been published [5, 24]. These recommend the use of specific calculators (or prediction models) to estimate the likelihood of treatment effectiveness and toxicity. While numerous prediction models have been published in recent years [25, 26, 27], few have been developed specifically for the older population, who may have different variables associated with survival or toxicity outcomes [28]. The use of assessment tools for functional status and non‐cancer life expectancy calculators is also recommended. Specific geriatric assessment tools may be useful to identify issues that may benefit from allied health input to support older patients through SOC treatment [16].

This study has some limitations. There was an incomplete recording of tumor p16 status in oropharyngeal cancer patients, limiting our ability to accurately evaluate the prevalence and impact of p16‐associated tumors. Furthermore, there was a nonrandom pattern of missing p16 data, with higher rates of missingness in earlier years of the cohort (Table S6). During this period, p16 testing was not universally funded (with patients required to pay for testing) and may have been influenced by sociodemographic and clinical factors, such as smoking greater than 10 pack years (which already places patients in a high‐risk category [29, 30]), introducing potential selection bias. In addition, our population included patients who had received RT for management of their mHNSCC. Information on patients receiving non‐RT management (e.g., surgery alone) was incomplete due to the nature of clinical practice and referrals to other centers. We were therefore unable to comprehensively evaluate patients receiving surgery as part of their cancer treatment. Beckett et al. reported that older patients with mHNSCC are less likely to receive adjuvant RT than younger patients [31], suggesting similar disparities in treatment approaches in surgically treated patients. Patients considered unfit or refusing RT, who received supportive care alone, were also not included in our analysis. These are more likely to be older patients, consistent with studies examining variations in RT utilization rates by age [32]. Due to the retrospective nature of this study, we were limited by data that was collected during clinical care; therefore, information from geriatric assessment tools or other functional assessments not performed during the study time period was unavailable. We assessed other variables as surrogate measures, for example, marital status and ECOG performance status, acknowledging that these are not as comprehensive as geriatric assessment tools. Lack of detailed information about specific patient comorbidities, social supports, and other issues discussed in MDT meetings is also a limitation to a more in‐depth examination of whether nonstandard treatment approaches were warranted or not. Comprehensive toxicity data, emergency presentations, and hospital admission data were also unavailable and, therefore, could not be analyzed for this study.

Our findings support SOC treatment approaches where appropriate for maximal disease control and survival. For older patients, though, this decision‐making is much more nuanced, as tumor control benefit needs to be considered in light of their biological age and balanced against their risk of treatment‐related toxicity and comorbidities, both of which may contribute to non‐cancer mortality. Patient preference and the presence of social supports are also important factors that may influence outcomes. Many older patients may not be candidates for SOC treatment approaches, and some variation in treatment approach is likely warranted. However, identifying areas of concern could allow the implementation of prehabilitation interventions before oncological treatment to better support selected older patients through SOC treatment [33, 34]. Due to the complexity in managing older patients, identifying potential challenges and establishing supports, before and during oncological treatment, could allow a greater proportion of older patients to receive SOC treatment approaches.

## Funding

This work was supported by the Australian Government Research Training Programme.

## Ethics Statement

Ethics board approval was obtained for this study from the SWSLHD Human Research Ethics Committee (Reference number: 2022/ETH01822).

## Consent

A waiver of consent was granted by the ethics board as per the NHMRC National Statement on Ethical Conduct in Human Research.

## Conflicts of Interest

Purnima Sundaresan has received an honorarium directed to Head & Neck Cancer Australia (a not‐for‐profit charity supporting patients with head and neck cancer) from Merck Sharp & Dohme for unrelated work and a speaker honorarium directed to TROG Cancer Research from Varian Medical Systems. Shalini Vinod has received honoraria from AstraZeneca, Roche, and Merck Sharp & Dohme, unrelated to this work. The other authors declare no conflicts of interest.

## Supporting information


**Table S1:** Standard of care definitions based on Australian EviQ guidelines.
**Table S2:** Univariable analysis of variables for standard‐of‐care treatment prescription, overall survival and progression free survival.
**Table S3:** Cause of death in palliative cohort.
**Table S4:** Multivariable analysis incorporating interaction term between age group and treatment strategy within the multivariable models for OS and PFS.
**Table S5:** Multivariable analysis of variables for survival outcomes (OS, PFS) for the oropharyngeal cohort (*n* = 354).
**Table S6:** Oropharyngeal cancer patient and tumor characteristics by availability of p16 status.

## Data Availability

The data that support the findings of this study are available on request from the corresponding author. The data are not publicly available due to privacy or ethical restrictions.
