# Supplementary material for: Variations in Treatment Prescription and Survival Outcomes Between Older and Younger Patients With Mucosal Head and Neck Squamous Cell Carcinoma Treated With Radiotherapy
Source: Head Neck. 2026 May 5;48(10):2837–48. doi: 10.1002/hed.70310 (PMC13595478; doi:10.1002/hed.70310)
Supplement: Supplementary file 1 [file HED-48-2837-s001.docx]

Supplementary table 1. Standard of care definitions based on Australian EviQ guidelines

| H+N subsite | Standard of care | Not standard of care |
| --- | --- | --- |
| **Glottic early stage**  Stage I-II (T1-2N0M0) | Chemotherapy- nil  RT regimens:   - 63Gy/28F (2.25Gy/F) - 60Gy/25F (2.4Gy/F) - 66-70Gy/33-35F (2Gy/F) - 65.25Gy/29F (2.25Gy/F) - 64-68Gy/32-34F (2Gy/F) - 55Gy/20F (2.75Gy/F) - 66Gy/30F (2.2Gy/F) |  |
| **Larynx + Hypopharynx** definitive EBRT w CT  Stage  *Larynx*: T3N0, T1-3 N1-3, consider unfavourable T2N0, consider T4 with no cartilage penetration and <1cm BOT involvement.  *Hypopharynx*: T3anyN, T1-2N1-3, consider unfavourable T2N0 | Chemotherapy:   - Cisplatin 100mg/m2, q21d - Cisplatin 40mg/m2, q7d   RT regimens:   - 66-70Gy/33-35F (2Gy/F) - 69.9Gy/33F (2.12Gy/F) | Chemotherapy:   - Cetuximab cycle 1- 400mg/m2 d1, cycle 2+ 250mg/m2 q7d - Carboplatin 4 AUC q21d - No chemotherapy |
| **Hypopharyx**  definitive EBRT  Stage I-II (T1-2N0M0) | Chemotherapy - nil  RT regimens:   - 70Gy/35F (2Gy/F) - 66Gy/30F (2.2Gy/F) |  |
| Oropharynx  definitive EBRT w CT  Stage anyTN2-3, T3-4anyN, select T2N1 | Chemotherapy:   - Cisplatin 100mg/m2, q21d - Cisplatin 40mg/m2, q7d   RT regimens:   - 66-70Gy/33-35F (2Gy/F) - 69.9Gy/33F (2.12Gy/F) |  |
| Oropharynx  definitive EBRT  Stage T1-2N0-1M0 | Chemotherapy - nil  RT regimens:   - 70Gy/35F (2Gy/F) - 66Gy/30F (2.2Gy/F) |  |
| Nasopharynx early stage  definitive EBRT  Stage T1 anyN M0 | Chemotherapy - nil  RT regimens:   - 66-70Gy/33-35F (2Gy/F) |  |
| Nasopharynx  definitive EBRT w CT  Stage T2+, anyN M0 | Chemotherapy:   - May have Induction CT - Cisplatin 100mg/m2, q21d - Cisplatin 40mg/m2, q7d   RT regimens:   - 70Gy/35F (2Gy/F) | Chemotherapy:   - Carboplatin + 5FU, q21d |
| Oral cavity, paranasal sinus + nasal cavity  definitive EBRT  Stage Unsuitable for surgery |  | Chemotherapy:   - Cisplatin 100mg/m2, q21d - Cisplatin 40mg/m2, q7d - Cetuximab   RT regimens:   - 70Gy/35F (2Gy/F) - 66Gy/30F (2.2Gy/F) |
| Paranasal sinus + nasal cavity  adjuvant EBRT +/- CT  Stage T3+, N+, positive margins  Some T1-2N0 w high risk features | Chemotherapy:   - Cisplatin 100mg/m2, q21d - Cisplatin 40mg/m2, q7d   RT regimens:   - 60Gy/30F (2Gy/F) - 66Gy/33F (2Gy/F) gross residual dx | Chemotherapy:   - Carboplatin + 5FU, q21d |
| Hypopharynx, larynx, oropharynx, oral cavity  adjuvant EBRT +/- CT  Stage high risk factors for recurrence | Chemotherapy:   - Cisplatin 100mg/m2, q21d - Cisplatin 40mg/m2, q7d   RT regimens:   - 60Gy/30F (2Gy/F) - 66Gy/33F (2Gy/F) gross residual dx |  |

Supplementary table 2. Univariable analysis of variables for standard-of-care treatment prescription, overall survival and progression free survival

|  |  | Standard of care treatment | | | Overall Survival | | | Progression free survival | | |
| --- | --- | --- | --- | --- | --- | --- | --- | --- | --- | --- |
| Variable | Strata | OR | 95% CI | P value | HR | 95% CI | P value | HR | 95% CI | P value |
| Age | <70 years | Ref |  |  | Ref |  |  | Ref |  |  |
|  | ≥70 years | 0.425 | 0.320-0.563 | **<0.001** | 1.780 | 1.482-2.137 | **<0.001** | 1.302 | 1.042-1.628 | **0.021** |
| Sex | Female | Ref |  |  | Ref |  |  | Ref |  |  |
|  | Male | 0.847 | 0.605-1.186 | 0.334 | 1.036 | 0.834- 1.286 | 0.749 | 0.952 | 0.744-1.218 | 0.694 |
| Marital status | Partnered | Ref |  |  | Ref |  |  | Ref |  |  |
|  | Unpartnered | 0.650 | 0.497-0.849 | **0.002** | 1.393 | 1.167-1.663 | **<0.001** | 1.112 | 0.907-1.362 | 0.308 |
| ECOG status | 0 | Ref |  |  | Ref |  |  | Ref |  |  |
|  | 1 | 0.474 | 0.353-0.636 | **<0.001** | 2.159 | 1.782-2.615 | **<0.001** | 1.821 | 1.452-2.285 | **<0.001** |
|  | 2+ | 0.283 | 0.176-0.455 | **<0.001** | 3.793 | 2.794-5.150 | **<0.001** | 2.448 | 1.677-3.573 | **<0.001** |
| Smoking status | Current | Ref |  |  | Ref |  |  | Ref |  |  |
|  | Former | 0.847 | 0.627-1.144 | 0.280 | 0.664 | 0.541-0.814 | **<0.001** | 0.853 | 0.683-1.067 | 0.165 |
|  | Never | 1.168 | 0.803-1.699 | 0.417 | 0.476 | 0.364-0.624 | **<0.001** | 0.672 | 0.502-0.901 | **0.009** |
| Tumour | Hypopharynx | Ref |  |  | Ref |  |  | Ref |  |  |
| site | Oropharynx | 1.491 | 0.916-2.427 | 0.109 | 0.470 | 0.344-0.643 | **<0.001** | 0.431 | 0.302-0.615 | **<0.001** |
|  | Oral cavity | 3.181 | 1.799-5.623 | **<0.0001** | 0.829 | 0.596-1.153 | 0.265 | 0.811 | 0.561-1.173 | 0.266 |
|  | Larynx | 2.240 | 1.299-3.862 | **0.004** | 0.596 | 0.426-0.834 | **0.003** | 0.459 | 0.309-0.682 | **<0.001** |
|  | Nasopharynx | 2.283 | 1.202-4.334 | **0.012** | 0.299 | 0.192-0.465 | **<0.001** | 0.417 | 0.262-1.664 | **<0.001** |
|  | Other | 1.442 | 0.610-3.409 | 0.404 | 0.776 | 0.435-1.385 | 0.392 | 0.777 | 0.415-1.457 | 0.432 |
| Tumour | I | Ref |  |  | Ref |  |  | Ref |  |  |
| stage | II | 0.603 | 0.243-1.493 | 0.274 | 1.324 | 0.794-2.209 | 0.284 | 1.352 | 0.766-2.386 | 0.299 |
|  | III | 0.274 | 0.120-0.624 | **0.002** | 1.537 | 0.976-2.420 | 0.064 | 1.460 | 0.857-2.487 | 0.165 |
|  | IVA/B | 0.249 | 0.113-0.549 | **0.0006** | 2.040 | 1.334-3.121 | **0.001** | 2.164 | 1.310-3.574 | **0.003** |
|  | IVC | 0.020 | 0.004-0.112 | **<0.0001** | 1.286 | 0.448-3.692 | 0.640 | 2.311 | 0.829-6.443 | 0.111 |
| SOC | Yes | NA |  |  | Ref |  |  | Ref |  |  |
|  | No | NA |  |  | 0.591 | 0.488-0.715 | **<0.001** | 0.536 | 0.432-0.665 | **<0.001** |

OR- odds ratio, HR- hazard ratio, CI- confidence interval, Ref- reference variable, SOC- standard-of-care

Supplementary table 3. Cause of death in palliative cohort

|  | Total cohort  n=199 | <70y  n=94 | ≥70yrs  n=105 | P value |
| --- | --- | --- | --- | --- |
| Deaths in palliative cohort, n | 157 | 79 | 78 |  |
| Cause of death, n (%) |  |  |  | 0.086 |
| Cancer related death | 130 (82.8) | 69 (87.3) | 61 (78.2) |  |
| Non-cancer death | 16 (10.2) | 8 (10.1) | 8 (10.3) |  |
| Unknown cause of death | 11 (7.0) | 2 (2.5) | 9 (11.5) |  |

## Supplementary Table 4. Multivariable analysis incorporating interaction term between age group and treatment strategy within the multivariable models for OS and PFS

|  |  | Multivariable analysis OS | | | Multivariable analysis PFS | | |
| --- | --- | --- | --- | --- | --- | --- | --- |
| Variable | Strata | Est | SE | P value | Est | SE | P value |
| Age | <70 years | Ref |  |  | Ref |  |  |
|  | ≥70 years | 0.438 | 0.174 | **0.013** | 0.381 | 0.192 | **0.049** |
| Sex | Female | Ref |  |  | Ref |  |  |
|  | Male | 0.026 | 0.120 | 0.827 | -0.030 | 0.134 | 0.825 |
| Marital status | Partnered | Ref |  |  | Ref |  |  |
|  | Unpartnered | 0.053 | 0.094 | 0.575 | -0.109 | 0.112 | 0.329 |
| ECOG status | 0 | Ref |  |  | Ref |  |  |
|  | 1 | 0.425 | 0.105 | **<0.001** | 0.327 | 0.123 | **0.008** |
|  | 2+ | 1.082 | 0.166 | **<0.001** | 0.622 | 0.219 | **0.006** |
| Smoking status | Current | Ref |  |  | Ref |  |  |
|  | Former | -0.418 | 0.116 | **0.001** | -0.154 | 0.125 | 0.217 |
|  | Never | -0.530 | 0.149 | **0.001** | -0.271 | 0.164 | 0.100 |
| Subsite | Hypopharynx | Ref |  |  | Ref |  |  |
|  | Oropharynx | -0.362 | 0.168 | **0.032** | -0.633 | 0.190 | **0.001** |
|  | Oral cavity | 0.233 | 0.183 | 0.204 | 0.120 | 0.199 | 0.548 |
|  | Larynx | -0.087 | 0.184 | 0.636 | -0.383 | 0.214 | 0.075 |
|  | Nasopharynx | -0.460 | 0.240 | 0.056 | -0.325 | 0.254 | 0.201 |
|  | Other | -0.033 | 0.308 | 0.915 | -0.063 | 0.328 | 0.849 |
| Tumour | I | Ref |  |  | Ref |  |  |
| stage | II | 0.416 | 0.273 | 0.133 | 0.276 | 0.295 | 0.351 |
|  | III | 0.598 | 0.251 | **0.019** | 0.306 | 0.280 | 0.275 |
|  | IVA/B | 0.914 | 0.243 | **<0.001** | 0.775 | 0.270 | **0.004** |
|  | IVC | 0.544 | 0.555 | 0.328 | 0.695 | 0.538 | 0.197 |
| SOC | No | Ref |  |  | Ref |  |  |
|  | Yes | -0.295 | 0.132 | **0.026** | -0.378 | 0.251 | **0.011** |
| Age:SOC | No interaction term | Ref |  |  | Ref |  |  |
|  | Interaction term | 0.069 | 0.216 | 0.750 | -0.390 | 0.251 | 0.122 |

Est- estimate, SE- standard error, Ref- reference variable, SOC- standard-of-care

## Supplementary Table 5. Multivariable analysis of variables for survival outcomes (OS, PFS) for the oropharyngeal cohort (n=354)

|  |  | Multivariable analysis OS | | | Multivariable analysis PFS | | |  |
| --- | --- | --- | --- | --- | --- | --- | --- | --- |
| Variable | Strata | HR | 95% CI | P value | HR | 95% CI | P value | |
| Age | <70 years | Ref |  |  | Ref |  |  | |
|  | ≥70 years | 2.439 | 1.459-4.078 | **0.001** | 1.724 | 0.969-3.068 | 0.069 | |
| Sex | Female | Ref |  |  | Ref |  |  | |
|  | Male | 1.590 | 0.848-2.978 | 0.152 | 1.461 | 0.683-3.123 | 0.332 | |
| Marital status | Partnered | Ref |  |  | Ref |  |  | |
|  | Unpartnered | 1.397 | 0.899-2.172 | 0.141 | 1.277 | 0.782-2.086 | 0.333 | |
| ECOG status | 0 | Ref |  |  | Ref |  |  | |
|  | 1 | 1.432 | 0.886-2.312 | 0.146 | 1.652 | 0.939-2.907 | 0.088 | |
|  | 2+ | 1.721 | 0.494-6.000 | 0.397 | 2.668 | 0.864-8.237 | 0.094 | |
| Smoking status | Current | Ref |  |  | Ref |  |  | |
|  | Former | 0.721 | 0.444-1.172 | 0.191 | 1.010 | 0.569-1.791 | 0.974 | |
|  | Never | 0.394 | 0.209-0.742 | **0.005** | 0.606 | 0.288-1.276 | 0.193 | |
| P16 | Negative | Ref |  |  | Ref |  |  | |
| status | Positive | 0.527 | 0.323-0.859 | **0.012** | 0.736 | 0.415-1.303 | 0.297 | |
| Tumour | I | Ref |  |  | Ref |  |  | |
| stage | II | 0.744 | 0.111-4.967 | 0.761 | 2.676 | 0.261-27.395 | 0.410 | |
|  | III | 1.410 | 0.376-5.296 | 0.612 | 2.146 | 0.258-17.872 | 0.483 | |
|  | IVA/B | 1.988 | 0.569-6.950 | 0.285 | 3.556 | 0.464-27.250 | 0.227 | |
|  | IVC | 1.875 | 0.271-12.996 | 0.526 | 4.833 | 0.373-62.569 | 0.233 | |
| SOC | No | Ref |  |  | Ref |  |  | |
|  | Yes | 1.167 | 0.682-1.997 | 0.575 | 0.975 | 0.551-1.724 | 0.930 | |

Supplementary Table 6. Oropharyngeal cancer patient and tumour characteristics by availability of p16 status

| Characteristic, n (%) | Full OPX cohort (n=570) | P16 status available (n=354) | P16 status missing (n=216) | P value |
| --- | --- | --- | --- | --- |
| Sex |  |  |  | 0.560 |
| Male | 477 (83.7) | 299 (84.5) | 178 (82.4) |  |
| Female | 93 (16.3) | 55 (15.5) | 38 (17.6) |  |
| Age group |  |  |  | 0.213 |
| <70 years | 471 (82.6) | 298 (84.2) | 173 (80.1) |  |
| ≥70 years | 99 (17.4) | 56 (15.8) | 43 (19.9) |  |
| Marital status |  |  |  | 0.076 |
| Partnered | 343 (60.2) | 224 (63.3) | 119 (55.1) |  |
| Unpartnered | 222 (38.9) | 128 (36.2) | 94 (43.5) |  |
| Missing | 5 (0.9) | 2 (0.6) | 3 (1.4) |  |
| Smoking status |  |  |  | **0.009** |
| Never | 140 (24.6) | 96 (27.1) | 44 (20.4) |  |
| Former | 218 (38.2) | 144 (40.7) | 74 (34.3) |  |
| Current | 181 (31.8) | 97 (27.4) | 84 (38.9) |  |
| Missing | 31 (5.4) | 17 (4.8) | 14 (6.5) |  |
| Median smoking pack years (IQR) | 30.0 (15.0-40.0) | 30.0 (12.0-40.0) | 39.5 (20.0-45.8) | **0.001** |
| Smoking pack years category^¶^ |  |  |  | **0.008** |
| <10 | 180 (31.6) | 129 (36.4) | 51 (23.6) |  |
| ≥10 | 309 (54.2) | 184 (52.0) | 125 (57.9) |  |
| Missing | 81 (14.2) | 41 (11.6) | 40 (18.5) |  |
| ECOG |  |  |  | **<0.001** |
| 0 | 381 (66.8) | 255 (72.0) | 126 (58.3) |  |
| 1 | 115 (20.2) | 63 (17.8) | 52 (24.1) |  |
| 2+ | 27 (4.7) | 8 (2.3) | 19 (8.8) |  |
| Missing | 47 (8.2) | 28 (7.9) | 19 (8.8) |  |
| Tumour stage |  |  |  | 0.941 |
| I | 16 (2.8) | 11 (3.1) | 5 (2.3) |  |
| II | 19 (3.3) | 12 (3.4) | 7 (3.2) |  |
| III | 99 (17.4) | 60 (16.9) | 39 (18.1) |  |
| IVA/B | 390 (68.4) | 249 (70.3) | 141 (65.3) |  |
| IVC | 7 (1.2) | 4 (1.1) | 3 (1.4) |  |
| Unknown* | 39 (6.8) | 18 (5.1) | 21 (9.7) |  |
| Grade (excluding NPX) |  |  |  | 0.401 |
| 1 | 33 (5.8) | 19 (5.4) | 14 (6.5) |  |
| 2 | 107 (18.8) | 77 (21.8) | 30 (13.9) |  |
| 3 | 147 (25.8) | 101 (28.5) | 46 (21.3) |  |
| 4 (undifferentiated) | 1 (0.2) | 1 (0.3) | 0 (0.0) |  |
| Missing | 282 (49.5) | 156 (44.1) | 126 (58.3) |  |
| Year of diagnosis |  |  |  | **<0.001** |
| 2010 | 43 (7.5) | 9 (2.5) | 34 (15.7) |  |
| 2011 | 62 (10.9) | 16 (4.5) | 46 (21.3) |  |
| 2012 | 61 (10.7) | 34 (9.6) | 27 (12.5) |  |
| 2013 | 60 (10.5) | 38 (10.7) | 22 (10.2) |  |
| 2014 | 61 (10.7) | 31 (8.8) | 30 (13.9) |  |
| 2015 | 64 (11.2) | 46 (13.0) | 18 (8.3) |  |
| 2016 | 73 (12.8) | 56 (15.8) | 17 (7.9) |  |
| 2017 | 84 (14.7) | 64 (18.1) | 20 (9.3) |  |
| 2018 | 62 (10.9) | 60 (16.9) | 2 (0.9) |  |

IQR- interquartile range, ¶ never smokers categorised as <10 pack years, * TNM stage incompletely recorded, NPX- nasopharynx cancer, OPX- oropharynx cancer
